# Hydrothermal epitaxy and resultant properties of EuTiO_3_ films on SrTiO_3_(001) substrate

**DOI:** 10.1186/1556-276X-9-266

**Published:** 2014-05-29

**Authors:** Fengzhen Lv, Jing Zhang, Cunxu Gao, Li Ma, Daqiang Gao, Shiming Zhou, Desheng Xue

**Affiliations:** 1 Key Lab for Magnetism and Magnetic Materials of the Ministry of Education, Lanzhou University, Lanzhou 730000, China; 2 Shanghai Key Laboratory of Special Artificial Microstructure Materials and Technology and School of Physics Science and Engineering, Tongji University, Shanghai 200092, China

**Keywords:** EuTiO_3_ films, Hydrothermal growth, Epitaxy, Multiferroics

## Abstract

We report a novel epitaxial growth of EuTiO_3_ films on SrTiO_3_(001) substrate by hydrothermal method. The morphological, structural, chemical, and magnetic properties of these epitaxial EuTiO_3_ films were examined by scanning electron microscopy, transmission electron microscopy, high-resolution X-ray diffractometry, X-ray photoelectron spectroscopy, and superconducting quantum interference device magnetometry, respectively. As-grown EuTiO_3_ films with a perovskite structure were found to show an out-of-plane lattice shrinkage and room-temperature ferromagnetism, possibly resulting from an existence of Eu^3+^. Postannealing at 1,000°C could reduce the amount of Eu^3+^, relax the out-of-plane lattice shrinkage, and impact the magnetic properties of the films.

**PACS:**

81.10.Aj; 81.15.-z; 61.05.-a

## Background

Interest in multiferroics has been recently revived, since coexistence and interactions of ferroelectric, ferromagnetic, and ferroelastic orderings in multiferroics [1-6] could be applied potentially to a range of novel multifunctional devices [6,7]. As one of the special multiferroic materials, EuTiO_3_ was found that in the bulk exhibits a *G*-type antiferromagnetic ordering below 5.3 K [8,9], and its epitaxial films transform into ferromagnetic under large enough lattice strain [10-13].

A variety of techniques are available to grow fine epitaxial perovskite films, such as pulsed laser deposition [11], molecular beam epitaxy [12], radio-frequency magnetron sputtering [14], and metal-organic chemical vapor deposition [15]. These methods share a common feature that high growth temperatures (>500°C) and costly equipments are usually necessary. In contrast, an attractive alternative technique for preparing epitaxial perovskite films is hydrothermal epitaxy [16-20], which allows direct deposition crystalline films using mild aqueous solutions at temperatures as low as 150°C [16,18] and avoids the research dependence on the costly aforementioned epitaxial growth equipments. In consideration of the merits of the hydrothermal epitaxy, however, nothing is currently known about the hydrothermal growth of epitaxial EuTiO_3_ films and their properties.

In this paper, we report the hydrothermal epitaxy of EuTiO_3_ films on SrTiO_3_(001) substrate at 150°C and the properties of the films. We find that the as-grown epitaxial EuTiO_3_ films show an out-of-plane lattice shrinkage and room-temperature ferromagnetism. Postannealing at 1,000°C evidences that this lattice shrinkage relates to the instabilities of Eu oxidation state in the films.

## Methods

The heteroepitaxial EuTiO_3_ films investigated were grown on SrTiO_3_(001) substrate by hydrothermal method. Prior to growth, a solution of KOH (10 M, 15 mL) was added into a suspension which was composed of TiO_2_ (0.2 g), Eu(NO_3_)_3_ · *x*H_2_O (1.0 g) and H_2_O (50 mL) with a subsequent constant stirring for 30 min. The resulting solution was then introduced into a 100-mL Teflon-lined stainless autoclave with a fill factor of 65%, where the SrTiO_3_(001) substrate was fixed inside. The autoclave was shifted to a preheated oven holding at 150°C. After 24 h of growth, the sample was removed from the autoclave, cleaned by deionized water, and then dried ready in the air for the subsequent measurements. The phase structure of the films was assessed by high-resolution X-ray diffractometry (HRXRD; Bede D1, Durham, UK). HRXRD longitudinal *ω*- 2*θ* scans were recorded with an analyzer composed of Ge channel-cut crystals, while a pole figure was taken in skew geometry and with open detector. To assess the morphology and microstructure of the films, the samples were cleaved into smaller pieces for investigation by scanning electron microscopy (SEM; Hitachi S-4800, Chiyoda-ku, Tokyo, Japan) and transmission electron microscopy (TEM; TecnaiTMG2F30, FEI, Hillsboro, OR, USA), the latter through the standard mechanical thinning and ion-milling processes. The elemental composition of the films was analyzed by X-ray photoelectron spectroscopy (XPS; Kratos AXIS Ultra^DLD^, Manchester, UK). The absence of water or hydroxyl in the films was evidenced by Fourier transform infrared spectroscopy (FTIR; Nexus870, Nicolet, Madison, WI, USA). The magnetic properties of the as-grown and annealed samples were measured in a superconducting quantum interference device magnetometry (SQUID). All magnetization data presented here are corrected for the diamagnetic background of the substrate. Postannealing of the as-grown sample was carried out in an Ar ambient for 10 h at 1,000°C.

## Results and discussion

**Figure 1:**
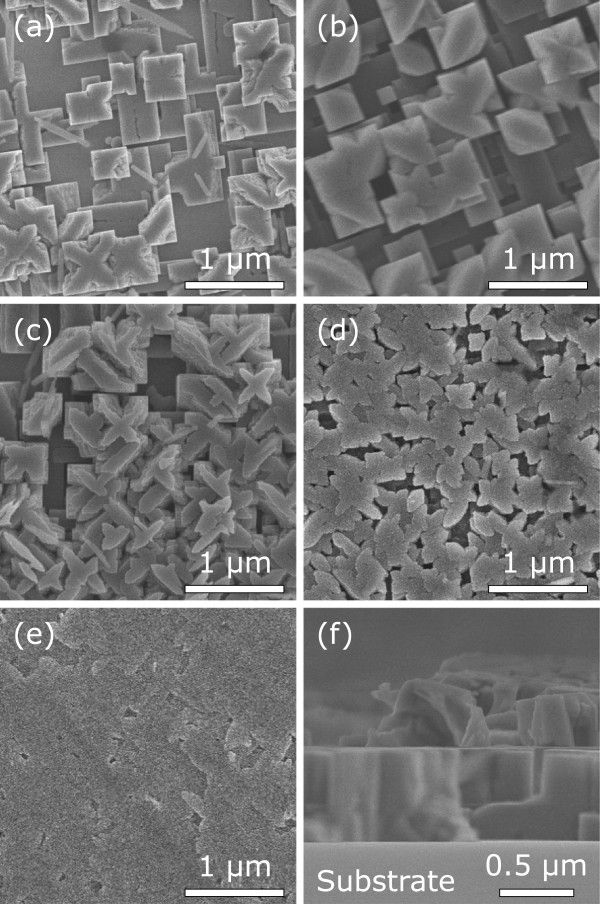
**Top-view and side-view SEM images.** Bird’s-eye view from the **(a)** edge, **(b)** near-edge, **(c)** middle-of-edge-and-center, **(d)** near-center, and **(e)** center of one sample surface. Note that the surface area of the SrTiO_3_(001) substrate is 5 × 5 mm^2^. **(f)** Cross-sectional SEM image taken in an arbitrary portion of the sample.

Most remarkable is the peculiar morphology observed by SEM from which a sequential growth of the films is proposed. Figure 1a,b,c,d,e displays the SEM images taken in the top view of one sample with surface graded from edge to center and the typical morphology of epitaxially overgrown and coalesced EuTiO_3_ films. Note that the surface area of the SrTiO_3_(001) substrate we used for growth is 5 × 5 mm^2^. We may indirectly visualize the growth evolution of the EuTiO_3_ films from the spacial morphological nonuniformity. As shown in Figure 1a, the existence of side facets observed at the top of micro-crystals reveals an initial nucleation growth in cross-like shape. The nucleation then processes from cross-shaped into tetragonal and after that into cuboidal. Accompanying the coalescence of cuboid in the first layer, nucleation on the second layer starts and develops, as shown in Figure 1b. Figure 1c,d clearly reveals the coalescence process of the micro-crystals on the second layer. A crisscross consisting of dense crosses shown in Figure 1c forms to coalesce the side facets of conjoined micro-crystals. Figure 1d shows coalescence of the crisscross on top of layers. The complete coalescence of the crisscross results in a great smooth surface of the films shown in Figure 1e. Interestingly, the crosses and the micron-sized tetragon develop regularly and orient highly, which reveals that the films are highly oriented and suggests a tetragonal structure of the film. This indication is evidenced by the following TEM and HRXRD results. Figure 1f shows a cross-sectional SEM image taken on an arbitrary portion of the sample. A layer with a uniform thickness of about 600 nm is clearly observed.

**Figure 2:**
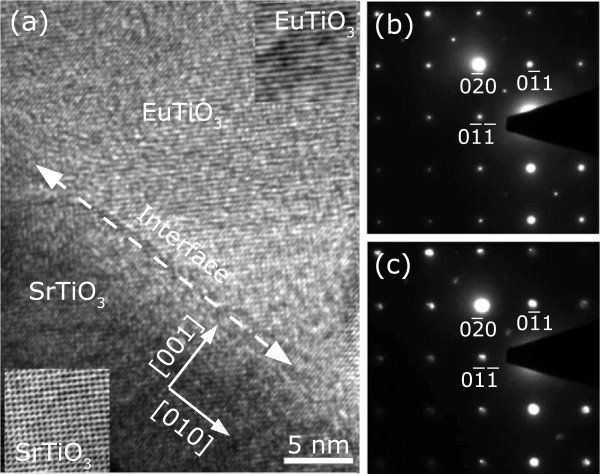
**High-resolution transmission electron micrographs and selected area electron diffraction patterns. (a)** Cross-sectional high-resolution transmission electron micrograph of the EuTiO_3_/SrTiO_3_(001) interface along the SrTiO_3_[ $\bar{1}00$] zone axis. The insets show the high-resolution micrographs of the EuTiO_3_ films and SrTiO_3_ substrate taken in focus, respectively. Selected area electron diffraction patterns of **(b)** EuTiO_3_ and **(c)** SrTiO_3_, respectively.

To directly investigate this peculiar epitaxial growth of the EuTiO_3_/SrTiO_3_(001) structure, the interface of the structure was examined by TEM. Figure 2a shows a cross-sectional high-resolution transmission electron micrograph of the EuTiO_3_/SrTiO_3_(001) interface along the SrTiO_3_[ $\bar{1}00$] zone axis. The lattice planes of the EuTiO_3_ film are clearly resolved and are found to be well ordered. Consecutive lattice planes at the interface between the film and the substrate is clear, which precisely and directly evidences a well epitaxial relationship between the deposited film and the substrate, although there might be few dislocations in the interface to release the internal stress due to slight lattice mismatch. The insets in Figure 2a show the high-resolution micrographs of the EuTiO_3_ films and SrTiO_3_ substrate taken in focus, respectively. Selected area electron diffraction (SAED) patterns of the films and substrate were also taken and are shown in Figure 2b,c, respectively. Both SAED patterns depict the identical crystallographic structure and indicate their epitaxial orientations with small lattice misfit and a highly oriented tetragonal structure of the film, which leads to a tetragonal surface morphology generally presented in nucleation developing stage, as shown in the aforementioned SEM images.

**Figure 3:**
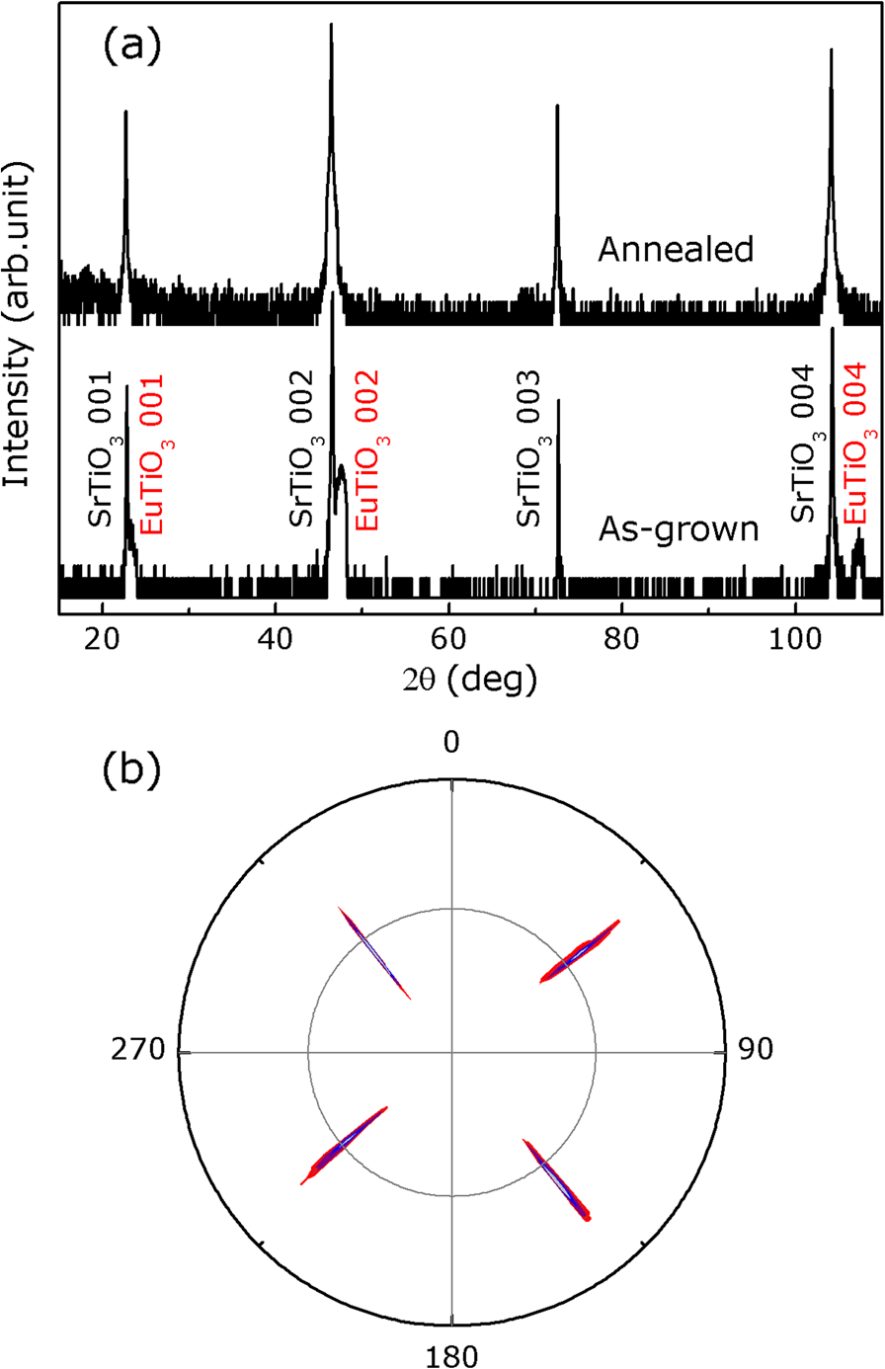
**HRXRD longitudinal scans and XRD pole figure. (a)** Symmetric HRXRD longitudinal *ω*- 2*θ* scans of the as-grown and postannealed EuTiO_3_ films on SrTiO_3_(001) substrate. **(b)** XRD {211} pole figure of the as-grown sample.

To investigate the crystallographic uniformity of this epitaxial growth, the EuTiO_3_/SrTiO_3_(001) structure was assessed by HRXRD. Both EuTiO_3_ and SrTiO_3_ were reported to have the cubic perovskite crystal structure at room temperature and have a lattice constant of 0.3905 nm [21], indicating zero lattice mismatch between EuTiO_3_ and SrTiO_3_. Figure 3a shows symmetric HRXRD longitudinal *ω*- 2*θ* scans taken within a 2*θ* range from 10° to 110° for the as-grown and postannealed samples. Apart from the (00*l*) (*l* = 1, 2, 3, and 4) reflections of SrTiO_3_, the (00*l*) reflections of EuTiO_3_ for the as-grown sample can be identified and no reflections pertinent to a secondary phase can be found, indicating that the epitaxial growth of EuTiO_3_ is oriented along the *c*-axis. The out-of-plane lattice constant of the as-grown films calculated from the (001), (002), and (004) peaks are 0.3789, 0.3821, and 0.3831 nm, respectively. They are much smaller than the reported value of 0.3905 nm for bulk EuTiO_3_[22,23] and show an out-of-plane lattice shrinkage of 2.9%, 2.1%, and 1.9%, respectively. The average shrinkage is 2.3%, which means that the out-of-plane lattice shrinks by about 2.3% along the *c*-axis. The in-plane epitaxial relationship between the films and the substrate was measured by azimuthal scans in skew geometry. Figure 3b shows an XRD {211} pole figure of the as-grown sample measured by setting 2*θ* = 57.92°. The reflections from EuTiO_3_ and SrTiO_3_ overlap in every streak measured by an azimuthal and sample-tilting angular scans. The in-plane fourfold symmetry of the EuTiO_3_/SrTiO_3_ orientation relationship is revealed by the four streaks in the pole figure, which shows an in-plane orientation relationship of EuTiO_3_〈100〉∥*SrTiO*_3_〈100〉. Evidently, the pole figure provides the same qualitative information as the SAED patterns, in that it reveals a fourfold symmetry and an excellent in-plane alignment of the EuTiO_3_ films and SrTiO_3_ substrate. Postannealing of the as-grown sample was carried out in an Ar ambient for 10 h at 1,000°C in order to compare the result with the report where the epitaxial EuTiO_3_ films were prepared by pulsed laser deposition [11]. Upon postannealing, symmetric HRXRD longitudinal *ω*- 2*θ* scans display that the EuTiO_3_ peaks shift toward lower angles and are superimposed on the SrTiO_3_ peaks without yielding any impurity phases, as shown in Figure 3a. It means that the out-of-plane lattice shrinkage of the as-grown films was relaxed by postannealing, possibly corresponding to the changes of oxidation state in Eu surroundings. It is reported that valence instabilities are an interesting and general phenomenon for rare earth ions in their compounds, for example, mixed valences, valence fluctuations, and surface valence transitions [24-27]. Our present work provides an opportunity to study further valence instabilities of Eu in EuTiO_3_ and their resultant properties.

**Figure 4:**
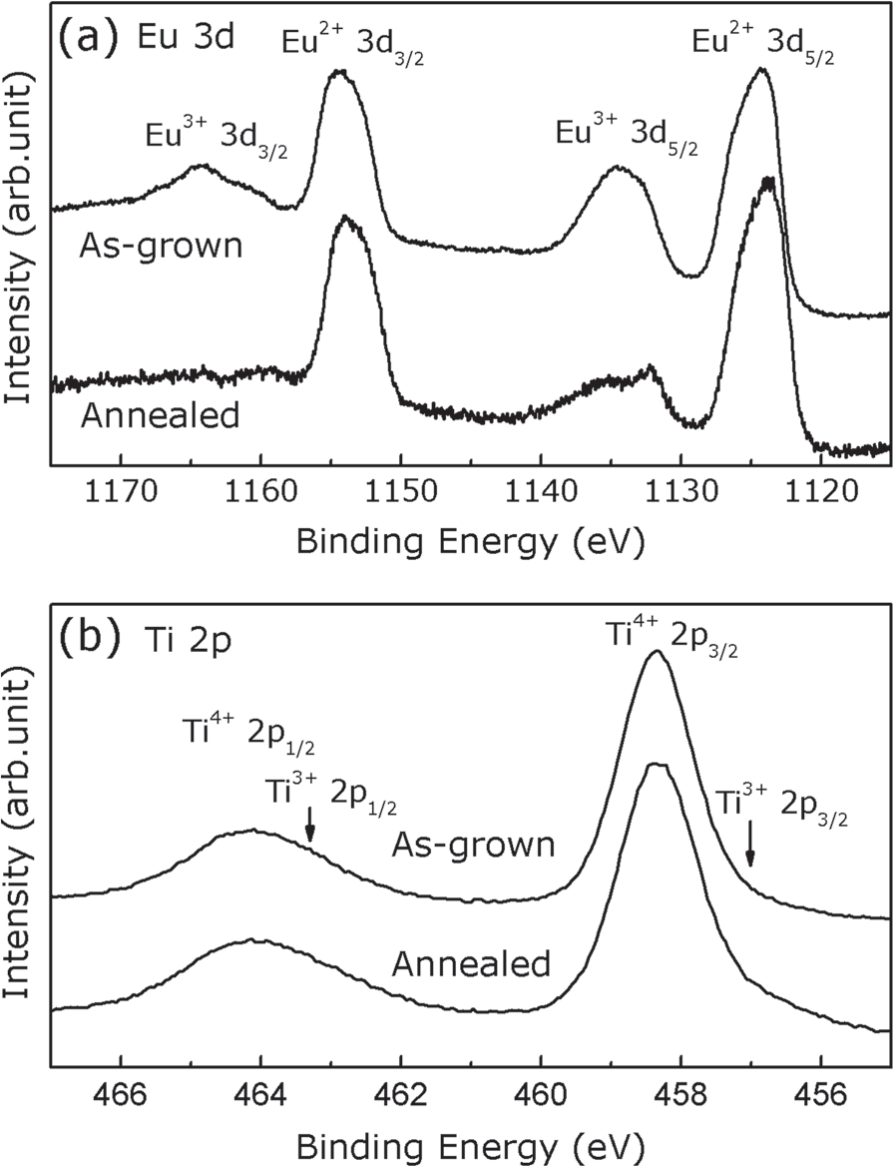
**XPS spectra of the as-grown and postannealed samples. (a)** A comparison of the Eu 3*d* core-level XPS spectra between the as-grown and postannealed samples. **(b)** Ti 2*p* core-level XPS spectra of the as-grown and postannealed samples.

The elemental composition of the films was then analyzed by XPS, which was taken within a binding energy scan range from 0 to 1,300 eV. No signals pertinent to K^+^ cation can be found, indicating that the films have no incorporation of K from the solvent. The Eu 3*d* and Ti 2*p* core-level XPS spectra of the as-grown sample are shown in Figure 4a,b, respectively. The results clearly exhibit that the as-grown sample consists of mixed Eu^2+^, Eu^3+^, and Ti^4+^ cations, in agreement with the peak positions of the cations shown in the XPS spectra from other studies [25-29]. The presence of Eu^3+^ indicates the necessity of anion excess in the as-grown films for charge balance and may affect the crystal lattice and magnetic properties of the films, which will be discussed later on. The Eu 3*d* core-level XPS spectra of the annealed sample are shown in Figure 4a, which reveals a reduction of Eu^3+^ quantity. The Ti 2*p* core-level XPS spectra of the annealed sample not only are dominated by the Ti^4+^ contribution but also plausibly exhibit the Ti^3+^ shoulders, as shown in Figure 4b. These results reflect a necessity to lose part of the ionic charge during the annealing process for charge compensation. Further investigations are necessary to understand the chemical details of the films and annealing process.

**Figure 5:**
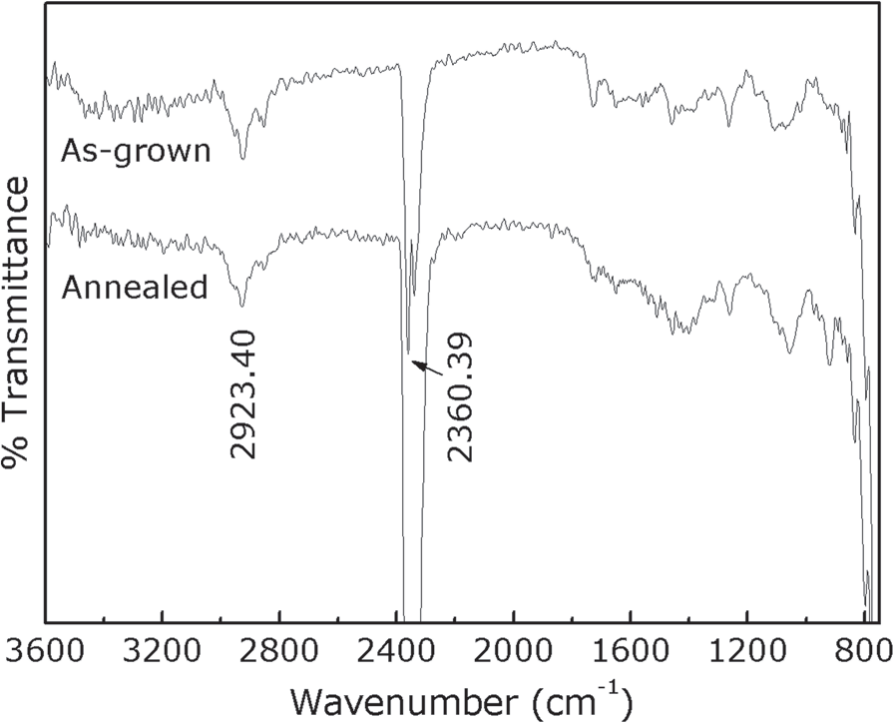
**FTIR spectra of the as-grown and postannealed samples.** The peak at 2,360.39 cm^-1^ is due to contributions from CO_2_ present in air.

It is important to realize the possible inclusion of water or hydroxyl in the as-grown films. Such issues have been reported in various perovskites prepared hydrothermally [30-32]. These impurities can contribute to charge balance in the as-prepared perovskites and be removed by annealing to produce defects, which when coupled with a metal can account for charge compensation [30,31]. Thus, our films were studied by FTIR. Figure 5 shows the FTIR spectra of the as-grown and postannealed samples for a comparison. No peaks pertinent to water or hydroxyl can be seen and resolved from the spectra; hence, the presence of water or hydroxyl and their resultant charge balance/compensation mechanisms are excluded in our films. The charge balance (compensation) in our as-grown (annealed) films is possibly made by oxygen excess (loss).

**Figure 6:**
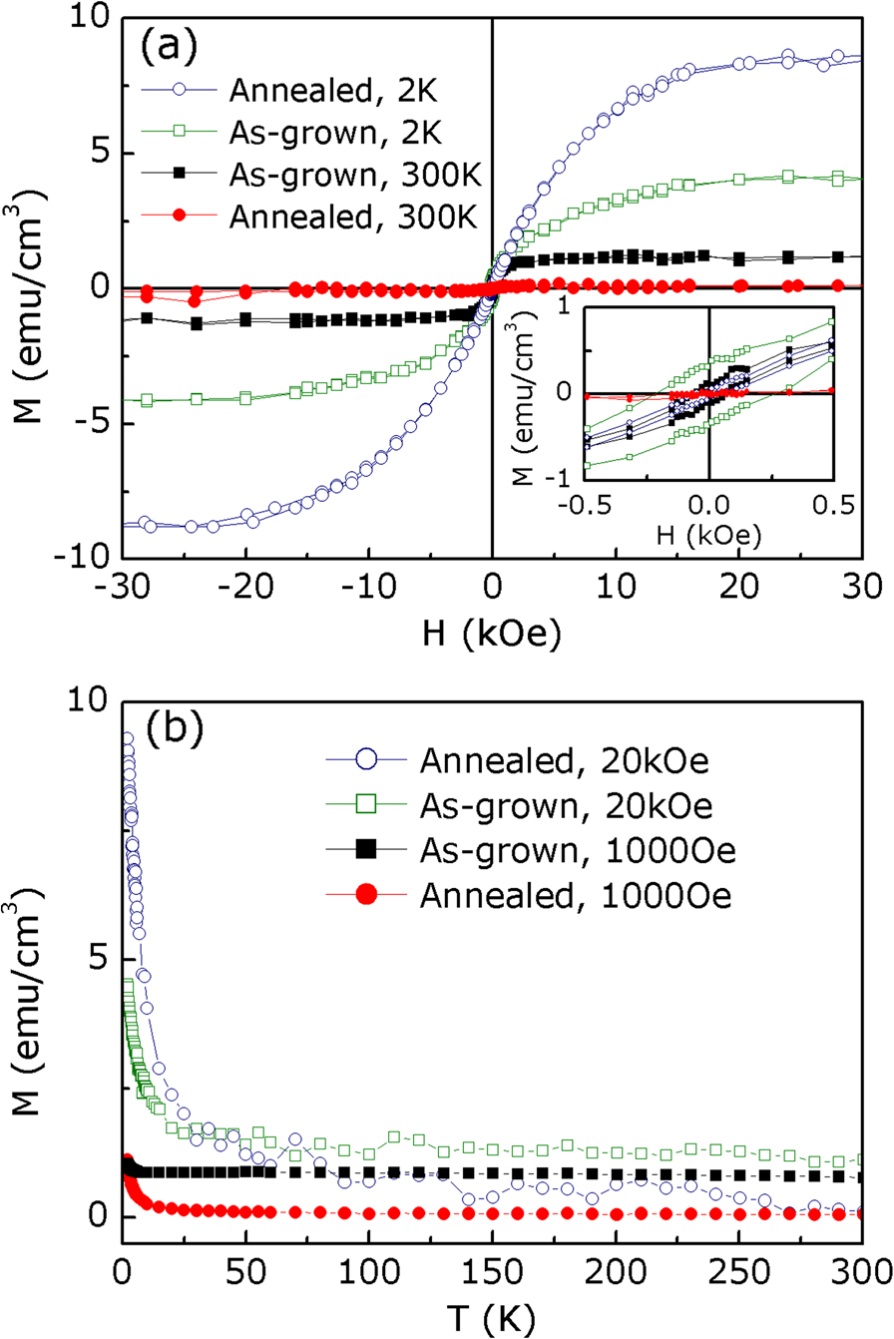
**Hysteresis loops and temperature dependence of magnetization. (a)** Hysteresis loops obtained at 300 and 2 K for the as-grown and annealed films with external field applied parallel to EuTiO_3_[100] direction. The inset magnifies the low magnetic field range. **(b)** Temperature dependence of the magnetization curves of the as-grown and annealed films at 1,000 Oe and 20 kOe external fields applied parallel to EuTiO_3_[100] direction.

Finally, we are interested in the magnetic properties of these films. The in-plane hysteresis loops for the as-grown films shown in Figure 6a were measured by SQUID with the magnetic field (*H*) parallel to the EuTiO_3_[100] direction at 300 K. The as-grown EuTiO_3_ films exhibit a ferromagnetic-like behavior. To quantitatively show the impact of the postannealing on its magnetic properties, the same piece of the sample after annealing was measured by SQUID to avoid errors from sample volume measurements. A striking decrease of *M*_S_ and a negligible ferromagnetic behavior for the annealed films are found and shown in Figure 6a. These results indicate that the oxidation states of Eu in the as-grown films provides magnetic moments and contributes to the magnetization. In order to get more information about the magnetism in these films, the in-plane hysteresis loops for the as-grown and annealed films were measured at 2 K. It can be seen from the loops shown in Figure 6a that both films exhibit a ferromagnetic behavior and an increase of *M*_S_ at 2 K. Surprisingly, the *M*_S_ value of the annealed films is much larger than that of the as-grown films at 2 K. It means that Eu^2+^ in the annealed films has magnetic contribution to magnetization at low temperature and implies that Eu^3+^ ion probably possesses less magnetic moment than Eu^2+^. Temperature dependence of the magnetization curves shown in Figure 6b compares the magnetic properties between the as-grown and annealed films in more detail. It clearly shows that the annealed films convert to ferromagnetic behavior as external magnetic field applied to the films is raised, implying the presence of a ferromagnetic phase transition in the annealed films at low temperature. Evidently, a thermal treatment of the as-grown films demonstrates obvious impact on their magnetic properties. Combining this result with that from XPS investigations, we can obtain that the valence instabilities of Eu in EuTiO_3_ films could result in the material being ferromagnetic at room temperature, which may extend the range and potential of this material for application.

## Conclusions

To summarize and conclude, using a hydrothermal method, EuTiO_3_ films with high crystalline quality were successfully grown on SrTiO_3_(001) substrate at a temperature of 150°C. The films show highly oriented and regularly shaped morphologies with graded spacial distribution, which reflects a sequential growth process of the films. Using this growth technique, EuTiO_3_ films grown on SrTiO_3_ substrate exhibit an out-of-plane lattice shrinkage, which could be relaxed by postannealing. Valence instabilities of Eu were found in the sample and result in the EuTiO_3_ films being ferromagnetic at room temperature, which provides an opportunity to study further their properties and potential applications.

## Abbreviations

FTIR: Fourier transform infrared spectroscopy; HRXRD: high-resolution X-ray diffractometry; SAED: selected area electron diffraction; SEM: scanning electron microscopy; SQUID: superconducting quantum interference device magnetometry; TEM: transmission electron microscopy; XPS: X-ray photoelectron spectroscopy.

## Competing interests

The authors declare that they have no competing interests.

## Authors’ contributions

FL carried out the synthesis and characterization of the samples, analyzed the results, and wrote the first draft of the manuscript. JZ participated in the design, preparation, and discussion of this study. CG contributed ideas for the growth of the samples and revised the manuscript. DX supervised the research. LM, DG, and SZ helped in the data acquisition of the samples and analysis. All authors read and approved the final manuscript.
